# On Instinct

**Published:** 1825-11

**Authors:** John Mason Good


THE LONDON
### Medical and Physical Journal.

No 4 OF VOL. LIV.] NOVEMBER, 1825. [No 321.

For many fortunate discoveries in medicine, and for the detection of numerous errors, the world is indebted to the rapid circulation of Monthly Journals; and there never existed any work, to which the Faculty, in Europe and America, were under deeper obligations, than to the Medical and Physical Journal of London, now forming a long, but an invaluable, series.—RUSH.

### ORIGINAL COMMUNICATIONS,
### SELECT OBSERVATIONS, &c.

ART. I.—*On Instinct.* By JOHN MASON GOOD, M.D. F.R.S. F.R.S.L.

THERE are certain actions, and trains of actions, occasionally to be met with among mankind, but more frequently and more strikingly among other animals, which indicate the employment of definite means to obtain a definite end, without the intervention of that chain of thought which constitutes reason; and which have, hence, been ascribed to a distinct principle, that has been distinguished by the name of INSTINCT.

Such, in the new-born infant, and indeed in the young of all mammalian animals, is the act of hunting out for the mother's milky food, and of sucking with a perfection which can never be acquired in subsequent life. Such is the whole process of nestling, or nidification, among birds; the periodical change of salt for fresh water among various fishes, as the salmon and the sturgeon, in the spawning season; and, among insects, the formation of the web, or exquisite decoy lines, of the spider, and the nice masonry of the bee and the termes *bellicosus*, or white ant.

The general fact admits of no dispute, though the modes of accounting for it have been various, and in the utmost degree unsatisfactory. It has been derived from mechanical powers,* from mental powers,† from both together,‡ and from an imaginary intermediate essence, supposed equally to pervade all embodied matter, and to give it form and structure.§ It has been made sometimes to include the sensations, sometimes the passions, sometimes the reason,|| and sometimes the ideas.¶ It

* DES CARTES; POLIGNAC, *Anti Lucret.*; KNIGHT, *Phil. Trans.* 1816.

† SMELLIE, *Philos. of Nat. Hist.* vol. i. 159. DARWIN, *Zoonom.* passim.

‡ REIMAR, Hamburg, 1769.

§ CUDWORTH, *Intellect. System*, 1743.

|| DARWIN, *Zoonom.* xvi. 2, 4, 13.

¶ CUVIER, *Annales du Museum d'Hist. Nat.* tom. xvi. 46.

has sometimes been restrained to animals, and sometimes extended to vegetable life.* The subject, therefore, in the present day, lies involved in no small degree of confusion; and even the term itself, by which the general fact to which it relates is expressed, that of *instinct*, or *instinctive power*, has been, and still continues to be, used in so loose and unsettled a sense, that there is not a Dictionary we can turn to, nor, perhaps, an interpretation we can form in our own minds, that presents us with a clear and definite idea upon its nature.

Under these circumstances, I beg to offer to the candid attention of the reader a few remarks, that may tend to give us a more exact and determinate illustration of the nature of the action, and, consequently, of what ought to be the extent and real meaning of the term.

It is well known, though it is necessary for one moment to advert to the fact, as a starting-post, that the visible or external world, by which we are surrounded, consists of bodies of two very different kinds: inorganic, or those possessed of no sort of internal organs for the production and support of peculiar functions; and organised, or those exhibiting such an arrangement. The first, destitute of vitality, are sometimes called dead matter; the second, endowed with it, are called living: and, to whichsoever we turn, we shall find, in the powers they possess, the changes they undergo, the laws to which they are subject, and the ends they accomplish, equal proofs of design and intelligence; and we shall find, also, that each of these divisions preserves its peculiar character, and only recognizes the system of regulations which distinctly apply to itself.

Thus, if we take up a stone, and examine in what it essentially differs from a plant or an animal, we shall find, on investigating the history of the stone, that it has been produced fortuitously; that it has grown by external accretion; and that it is only destructible by chemical or mechanical means. While, on investigating the history of the plant or the animal, we shall find it to have been produced by generation; to have grown by nutrition, or internal instead of external, accretion, and to be destructible by death; to be actuated by an internal power, and possessed of parts mutually dependent, and contributory to each other's functions. In what this internal power consists, we know not: it has been denominated a *vis insita*, a *vis vitæ*, an *archæus*, or, more correctly, an ἀρχάις Φύσις, which is the language of Hippocrates;—it has been ascribed to thermogen, caloric, or the elementary principle of heat, to oxygen, to electricity, or the voltaic aura;—all which, however, are conjectures, and nothing more, and give us no definite idea of the

---

* Lamarck, *Philosophie Zoologique.*

nature of the principle itself. We know it to be a distinct power from that of thought or of sensation;—we trace it, under different modifications, in plants as well as in animals; in living fluids as well as in living solids, as we shall notice presently; in the sap or the blood, as well as in the bud or the muscle;—we behold it running co-extensively with life itself, from its first germ to its last decay; from the seed, or the egg, to the stroke of death; from the slenderest fibre to the most complicated organ;—and we hence regard it as the source or principle of life.

Now, if we examine accurately, we shall find that what physiologists and pathologists have thus traced out and denominated a *living principle,* psychologists and moral philosophers have referred to under the name of an *instinctive principle,* whenever they have treated of, or glanced at, this last as a simple power or essence, and have not confounded it with intelligence or sensation. Or, to speak somewhat more strictly, they have regarded it as the living principle, manifestly and immediately directing its exertions towards the health, preservation, or reproduction of a vital frame, or any part of such frame.

In this view of the subject, the two distinct terms become co-ordinates. The law of instinct is the law of the living principle; instinctive actions are the actions of the living principle, and either is that power which characteristically distinguishes organised from unorganised matter, and pervades and regulates the former as gravitation pervades and regulates the latter: uniformly operating, by definite means, in definite circumstances, to the general welfare of the individual system, or of its separate organs; advancing them to perfection, preserving them in it, or laying a foundation for their reproduction, as the nature of the case may require. It applies equally to plants and to animals, and to every part of the plant as well as to every part of the animal, so long as such part continues alive. It is this which maintains from age to age, with so much nicety and precision, the distinctive characters of different kinds and species, which carries off from every part of an individual system its waste or worn-out matter,—supplies it with new, and, in a thousand instances, suggests the mode of cure, or even effects the cure itself, in cases of injury or disease, which is what physicians mean by the term *vis medicatrix naturæ;* and what is popularly referred to, when we speak of leaving a disease to nature. It is hence the strawberry travels from spot to spot, and the cod or the cuckow from shore to shore, or from climate to climate. Of its cause or nature, we know no more than we do of the cause or nature of gravitation; and every hypothesis that has been offered upon the subject has only served to perplex it; and to involve its inventor in a wearisome and fruitless disputation. It is neither essential mind nor essential matter; it is neither passion nor sensation; but, though unquestionably distinct from all these, is capable, as we shall see presently, of combining with any of them. It is possessed of its own book of laws, to which, under the same circumstances, it adheres, without the smallest deviation; and, as already observed, its sole and uniform aim, whether acting generally or locally, is that of health, maturity, or reproduction.

In supplying the place of reason, it is perpetually assuming its semblance. Let us take a plain example or two from both the vegetable and the animal worlds.

In order that the seeds of plants should produce and perfect their respective kinds, it is necessary that their shoots should rise to the surface of the earth, to enjoy the benefit of light and air. Now, in whatever direction the eye of a seed, from which germination first radiates, is placed, these shoots ascend equally to the surface, either in curved or straight lines, according as such ascent may be most easily accomplished. Mr. John Hunter sowed a quantity of peas and beans, with their eyes placed in different directions, in a tub, which he afterwards inverted, so that the bottom was turned uppermost, while the mould was prevented from falling out by a fine net; and, in order that the under surface might possess a superior stimulus of light and heat to the upper, he placed looking-glasses around the mouth of the tub, in such a way that a much stronger light was reflected upon the inverted mould than that of the direct rays of the sun; while, at the same time, he covered the bottom of the tub with straw and mats, to prevent the mould, in this direction, from being affected by solar influence. Yet the same instinctive law of ascent still prevailed. After waiting a considerable length of time, and perceiving that no shoots had protruded through the lower surface of the mould, he examined the contents of the tub, and found that they had all equally pressed upwards, and were making their way through the long column of mould above them towards the reversed bottom of the vessel; and that, where the eyes had been placed downward, the young shoots had turned round, so as to take the same direction.

As one experiment leads on to another, he determined to try the effect of placing other seeds of the same sorts in a tub to which a rotatory motion should be given, so that every part of it might be equally and alternately uppermost, and the seeds should have no advantage in one direction over another. Here, as we often behold in other cases, the instinctive principle of accommodation was baffled by a superior power; and the different shoots, instead of ever turning round, uniformly adhered to a straight line, except where they met with a pebble or any other resistance, when they made a curve to avoid such obstruction, and then resumed a straight line in the direction in which they were hereby thrown, without ever endeavouring to return to the original path.

Among animals, we have numerous proofs of a like impulse; and we have also proofs of its being occasionally overpowered by a stronger cause. Thus, in cases of eruptive fever, there is an obvious effort of the instinctive principle to throw the morbific matter towards the surface of the body, where it can do least mischief; and, when a deep-seated abscess has formed in the immediate neighbourhood of a cavity that cannot be opened into without great danger, the same instinctive principle of preservation leads forward the action in a different direction; though, as in the experiment of the bean-seeds in the inverted tub, with much greater labour and difficulty, and the abscess at length opens externally; and the remedial process of the formation of new living matter, which immediately succeeds, commences under the same mysterious guidance. In the discharge of extra-uterine fœtuses, the same skilful influence is sometimes still more striking; and if, in the course of this common tendency to the surface, an obstructive cause be encountered, of superior force to the instinctive principle itself, the latter, as in the experiment of the beans exposed to the action of a rotatory motion, is overpowered, and the result is doubtful, and often fatal.

But these examples are general: let us advert to a few of a more particular nature.

All the different species of birds, in constructing their nests, not only adhere to a peculiar plan, but, whenever they can obtain them, a peculiar kind of materials. Yet, if these materials cannot be procured, the accommodating power of the instinctive principle, as i[illegible]e cases just related, directs them to others, and suggests the best substitutes. Thus, the redbreast uniformly prefers oak-leaves as a lining for her nest, whenever she can acquire them; but, if these be not to be had, she supplies their place by moss and hair. So, where the bird of any kind, about to lay her eggs, is of small size, and the eggs are more than commonly numerous, the nest is always made proportionably warm, that the nestlings may all equally partake of the vivifying heat. Thus the wren, which lays from ten to eighteen eggs, constructs her little edifice with the greatest care, and of the warmest materials; while the plover and the eagle, whose eggs are so few that the body of the mother may easily cover them, build with little solicitude, and sometimes content themselves with the naked cleft of a rock. And thus, too, in very cold winters in Lapland, the fond water-fowl will occasionally strip the down off its breast, to line its nest and protect its progeny.

"When a wasp, in attempting to transport a dead companion from the nest, finds the load too heavy, he cuts off its head, and carries it out in two portions."*

Such is the accommodating power of the instinctive principle in animals, when directed to a particular purpose. Nor are the examples of it fewer or less striking in the vegetable world.

A strawberry offset, planted in a patch of sand, will send forth almost the whole of its runners in the direction in which the proper soil lies nearest; and few or none in the line in which it lies most remote.

"When a tree which requires much moisture," says Mr. Knight, "has sprung up or been planted in a dry soil in the vicinity of water, it has been observed that much of the larger portion of its roots has been directed towards the water; and that, when a tree of a different species, and which requires a dry soil, has been placed in a similar situation, it has appeared, in the direction given to its roots, to have avoided the water and moist soil."†

"When a tree," remarks Dr. Smith, "happens to grow from seed on a wall, (and he particularly alludes to an ash, in which the fact actually occurred,) it has been observed, on arriving at a certain size, to stop for a while, and send down a shoot to the ground. As soon as this shoot was established in the soil, the tree continued increasing to a large magnitude."‡

The best means, perhaps, that a plant can possess of resisting the effects of drought is a tuberous or bulbous root. The grass called phleum *pratense*, or common cat's-tail, when growing in pastures that are uniformly moist, has a fibrous root, for it is locally supplied with a sufficiency of water. But in dry situations, or such as are only occasionally wet, its root acquires a bulbous form, and thus instinctively accommodates the plant with a natural reservoir. And there are various other grasses, as the alopecurus *geniculatus*, or geniculate fox-tail, that exhibits the same curious adaptation.§

There are some philosophers and physiologists who have ascribed the whole of these very extraordinary phænomena to the mechanical powers of gravitation and centrifugal force. Among these I may especially mention Des Cartes, in the middle of the seventeenth century, and the Cardinal de Polignac‖

---

* *Memoirs of* SMELLIE, vol. ii. REAMUR, tome xi. p. 241. See, for other curious examples, the Swedish *Amœnitates Academicæ*, vol. iii. art. 45, Noxa Insectorum; M. A. BÆCHNER, 1752; and compare with the younger HUBER'S *Recherches sur les Mœurs des Fourmis indigènes.*

† *Phil. Trans.* 1811, p. 210.

‡ *Introduction to Botany*, p. 114. § *Id.* p. 41, 113.

‖ *Anti-Lucretius, sive de Deo et Natura*, libri Novem. 1747.

towards its close, who applied this principle of action equally to plants and brute animals; and Mr. Knight in our own day,* who seems disposed to confine its operation to vegetables, leaving us to account for the same phænomena in brutes as we may be able.

There are others, as already hinted at, who ascribe them equally to an intelligent principle, and sentient passions and emotions, operating in plants as well as in animals; among whom, more especially, was Dr. Darwin.† Of these two causes, the instances here selected, (and thousands of others might be added to them,) sufficiently prove that the first is inadequate, and that the second does not exist: at least, that the phænomena are found in organised forms, in which, to a certainty, the precise organs do not exist which are the only known seats of intelligence and sensation in the visible world,—as the brain and the nerves. They are hence to be resolved into another cause equally remote from either, apparently more complex in its operation than that of gravity, but probably less so than those of intelligence and feeling; embracing a distinct family of well-defined and cognate actions, always aiming at the same common end, the perfection, preservation, or reproduction of the system in which they exist; and constitute what we should denominate INSTINCT, the general property of the living principle, or the law of organised life.

Instinct, sensation, and perception, are principles essentially different: they may, indeed, exist conjointly, but each of them is capable of existing separately. Instinct is the common law of living organised matter, as gravitation is of unorganised; and the former bears the same analogy to sensation and perception, as the two latter do to crystallisation and chemical affinity. Instinct is the general faculty of the organised mass, as gravitation is of the unorganised mass; sensation and perception are peculiar powers or faculties appertaining to the first, as crystallisation and affinity are appertaining to the second: they can only exist under certain circumstances of the organised or unorganised matter to which they respectively belong.

This parallel, indeed, may be carried much further. Gravitation discovers itself under different modifications, different degrees of power, and consequently different effects. Instinct evinces an equal diversity in all these instances. Gravitation belongs equally to the smallest and to the largest portions of unorganised matter: instinct, in like manner, belongs equally to the smallest and to the largest portions of organised matter, so long as it continues alive: it exists alike in solids and in

---

* *Phil. Trans.* 1811, 1816.

† *Phytologia*, and *Botanic Garden*, passim.

fluids,—in the whole frame, and in every part of the frame,—in every organ, and in every part of every organ. Sir Isaac Newton established the doctrine of gravitation, and overcame all objections to it, chiefly by the modesty with which he propounded and illustrated it. Without pretending to determine any thing concerning its substance or essence, he contented himself with recognising it by its laws and operations. It is the aim of the present attempt to follow this great example, and to tread in the same humble path; and, leaving all discussions concerning the essential nature of instinct or of organised life, on which instinct is dependent, and which constitutes its sphere, as matter constitutes the sphere of gravitation, to point out nothing more than the nature of its action, and occasionally to catch a glance at the laws by which it is regulated.

From what has already been said, however, it is obvious that the influence of instinct runs equally through the whole range of vegetable as well as of animal life; and consequently that its powers or principles, whatever they consist in, are essentially different from those of perception and sensation. Instinct is the common property of organised life in all its forms; but life itself is not necessarily connected either with reason or sensation: and it is of no small consequence that we attend to this curious and extraordinary fact, the proofs of which are abundantly in our possession. Mr. John Hunter has sufficiently shown the blood to be alive, though a fluid, and to have all the common properties of life, as irritability, contractility, and a power of maintaining its natural scale of heat, whatever be the temperature of the surrounding atmosphere.* It is, indeed, perpetually evincing its attachment to life, by a due and discretionary exercise of these properties with a view of preserving life, and consequently by a clear manifestation of an instinctive faculty. It resists equally every excess of cold or of heat, that may be injurious to it; and hence sometimes raises the thermometer, and sometimes depresses it, so as to maintain a healthy equilibrium. It contracts itself, like the muscular fibre, upon the application of an appropriate stimulus, and conveys the principle of life, and powerfully assists in applying that stimulus, to parts in which the vital action is languid, or has altogether ceased. There is no part of the living system that displays, in a more eminent degree, the faculty of self-preservation, or self-production, of attachment to life, or of resistance to whatever is injurious, than the blood; and yet every one must perceive that this faculty is pure unmixt instinct, equally destitute of feeling or intelligence.†

* *On Blood, &c.* p. 20, and following.

† See *Mémoires sur l'Irritabilité considerée comme Principe de Vie dans la Nature organisée.*—Girtanner, Journ. de Phys. 1790.

I have said, however, that the same principle appears to exist in the fluids of plants; and Mr. Knight appears to have established this by facts, which are equally surprising and satisfactory: for he has shown that the sap of potatoe and of mint-leaves, and that of the leaves and shoots of the vine, when plunged into a moist and warm soil, are disposed, like the blood, to give rise to vascular or organic forms, and to produce bulbs or roots, more or less perfect; and give evident proofs of an effort to preserve and extend life,* and, consequently, of an instinctive power,—of that power which consists, in every instance, accordantly with the definition of instinct already given,—in a simple operation of the principle of organised life, by the exercise of certain natural powers directed to the present or future good of the individual.

In like manner, the bones, the cartilages, the cellular membrane, and even the cuticle, are alive; but, in a state of health, they are as destitute of sensation, as well as of intelligence, as the blood; and, whether in a state of health or of disease, are always destitute of the latter; though, by a variety of morbid actions, the former is often produced in all of them. So, in the new-laid egg, we have an equal proof of an instinctive principle, —of the same faculty of self-preservation, of the same attachment to life, and resistance to destruction. For, like the blood of a healthy individual, the new-laid egg, whose vessels are merely in a nascent state, and which can scarcely be regarded otherwise than as a fluid, is capable of equally counteracting cold, heat, and putrefaction; and does forcibly counteract them for a considerable period longer than an egg that has been frozen, or in any other way deprived of its vital and instinctive power.

Sensation and perception, so far as we are capable of witnessing, can only exist in appropriate organs, as the nerves, or modifications of nerves, which are the only known seat of the one; and the brain, or some modification of the brain, which is the only known seat of the other; and hence, wherever these organs, or either of them, be discoverable, it is consistent with right reason to infer that the faculty also exists to which they respectively give rise. But, on the contrary, when neither of these organs exist, as in plants, and a multitude of the lowest tribes of animals, (which, in the zoological system of Lamarck, are on this account denominated *apathic*, or insentient,) we have the same reason for inferring that, though life is present, and indeed in many instances, peculiarly tenacious and vigorous, there is neither intelligence nor sensation; and that the whole of their vital functions and operations are performed by the

* *Phil. Trans.* 1816, p. 289.

common law of instinct, which, acting in different ways in different organs and beings of different structures, appertains to living matter of every kind; which is equally independent of nerves and brain, of sensation and perception; which requires no tuition or discipline, admits neither of intensity or dulness; but is perfect and mature from the first, and uniform in its mode of action from infancy to old age.

Is there, then, no such thing as instinctive feeling? a term in every one's mouth, and which every one, till he tries, thinks that he comprehends? What but an instinctive feeling is the love of life, the dread of death? the economy of pairing, and the desire of progeny?

Wherever feeling at all exists, these, in a certain sense, may unquestionably be called instinctive feelings. But it should be remembered that the expression is, in every instance, of a compound character, and involves two distinct ideas, which may exist either separately or conjointly; and that we have the same reason for using the phrase *instinctive intelligence*, as *instinctive feeling;* for we can only mean, or ought only to mean *instinct* combined with *intelligence*, or *instinct* combined with *feeling*, according to the nature of the case before us.

Combinations of this kind, indeed, are not unfrequent, and I shall presently offer examples of them; but it becomes necessary to observe, antecedently, that all the operations we are now adverting to, and which are usually characterised as instinctive *feelings*, as self-preservation, attachment to life, resistance of destruction, reproduction of the whole or of separate parts of the system, and even the economy of pairing, though often united with feeling, and not unfrequently with intelligence as well, occur, nevertheless, in a multiplicity of instances in which we have either direct proofs or the most cogent reasons for believing that there is neither feeling nor intelligence whatever, and that the whole is the result of pure, unintelligent, insentient instinct.

Whence derive the young of every kind a knowledge of the peculiar powers that are to appertain to them hereafter, even before the full development of the organs in which those powers are to reside? To adopt the beautiful language of the first physiologist of Rome,

> Cornua nata prius vitulo quam frontibus exstent,
> Illis iratus petit, atque infestus inurguet:
> At catulei pantherarum, scymneique leonum,
> Unguibus, ac pedibus jam tum morsuque repugnant,
> Vix etiam quom sunt dentes unguesque createi.
> Alitum proporro genus alis omne videmus
> Fidere, et a pennis tremulum petere auxiliatum.*

* LUCRET. *De Ner. Nat.* v. 1033.

The young calf, whose horns
Ne'er yet have sprouted, with his naked front
Butts, when enrag'd; the lion-whelp or pard
With claws and teeth contends, ere teeth or claws
Scarce spring conspicuous; while the pinioned tribes
Trust to their wings, and, from th' expanded down
Draw, when first fledged, a tremulous defence.

In like manner, an infant, in danger of falling from its nurse's arms, stretches out its little hands to break the fall, as though informed by experience of the use of such an action. In all these cases we have instances of pure instinct; but the same conduct is pursued in adult age, and affords us examples of instinct combined with a higher or lower degree of intelligence, and intelligence which, instead of opposing the instinctive exertion, encourages and fortifies it.

So, when caterpillars are shaken from a tree, in whatever direction they descend, they all instantly turn towards the trunk and climb upwards, though till now they have never been on the surface of the ground, and are directed by instinct alone as to the track they are to pursue.

In plants, we meet with examples of the same kind, and equally marvellous. Thus, the stalk of the convolvulus twines from the left or east by the south to the west, the face being towards the south; the phaséolus *vulgaris*, or kidney-bean, pursues the same course; while the honey-suckle and the hop take a perfectly reverse direction; the same sun, the same soil, the same atmosphere, operating on all of them at the same time. The instinctive power is obvious, as is also the absence of both sensation and reason. But who will reveal to us the cause of this difference?

In the following instances the cause is obvious: it proceeds from the peculiar structure and powers of the different animals to which they relate; and it would, perhaps, be as obvious to us in the preceding, were we as intimately acquainted with the nature of plants as of animals. The squirrel, the field-mouse, and the very curious bird called the nut-hatch (sitta *Europœa)*, live equally on hazle-nuts, as their most delicious food; but each of them opens the kernel in a very different manner. The squirrel, after rasping off the small end, splits the shell in two with his long fore-teeth, as a man does with his knife. The field-mouse nibbles a hole with his teeth, as regularly as if drilled with a wimble, and yet so small that it is wonderful how the kernel can be extracted through it: while the nut-hatch picks an irregular hole with his bill; but, as this artist has no paws with which to hold the nut while he pierces it, like an adroit workman he fixes it in some cleft of a tree or in some crevice, which answers the purpose of a vice; when, standing over it, he readily perforates the stubborn shell, and, while at work, makes a rapping noise, that may be heard at a considerable distance.*

In all these cases the same action is perpetually pursued, without the slightest change, from generation to generation, and hence proves itself to be simple unmixt instinct. It is, indeed, accordant with the dictates of intelligence; and hence Darwin, Smellie, and some other physiologists, have conceived that these two principles are of the same character, and that the one has given rise to the other. For the same reason, Dr. Darwin asserts that, in the promptitude and perfection with which the new-born infant seeks out and sucks its mother's breast, although the chain of thought which directs it to the accomplishment of its object is concealed from view, it still exists, and he endeavours to follow it up and develope it. In this, however, it is not worth while to accompany him; for the whole process, even upon his own showing, is so complex, that it would rather require the genius of an adult Newton to unfold it, than yield to the dawning powers of a new-born infant: while, in thousands of other cases of the instinctive faculty, the most excursive theorist cannot picture to his imagination any thing like a chain of thought or previous reasoning, nor even any thing like habit or imitation, by which the means and the end are joined together.

Let us take, as an example, the very common instance of a brood of young ducks, brought up under a hen, and, contrary to all the instincts and feelings of the foster-mother, plunging suddenly into the water, while she herself trembles piteously on the brink of the pond, not daring to follow, and expecting every moment to see them drowned. By what kind of experience or observation,—by what train of thought or reasoning, has the scarcely-fledged brood been able to discern that a web-foot fits them for swimming, and that a fissured foot would render them incapable? a knowledge that mankind have only acquired by long and repeated contemplation, and which has never been fully explained to this hour. Habit, imitation, and instruction, would all concur in teaching them to flee from the water, as a source of inevitable destruction; and yet, in opposition to all these influences and premonitions, we see them rush into it, and harmlessly: we see them obeying an irresistible impulse, which directs them to what is fitting, and which is stampt in the interior of their little frames, and an impulse equally remote from the laws of mind and of mechanism.

In like manner, by what process of imitation, education, or reasoning, does the nut-weevil (curculio *nucum)*, whose little

---

* White's *Nat. Hist. of Selborne.*

larve, or maggot, is familiar to all of us, from its being found so frequently in the kernel of the hazel-tree, seek out exclusively, and with the nicest knowledge of the plant, the green hazel in the month of August, while its nut-shell is yet soft and easily penetrable? What past experience or course of argument instructs her that this is the fruit best adapted, or perhaps only adapted, to the digestive powers of its future progeny? With a finished knowledge of her art, as soon as she is sufficiently pregnant to deposit her eggs, she singles out a nut, pierces it with her proboscis, and then, turning round with an accurate adjustment, drops an egg into the minute perforation; having accomplished which, she passes on, pierces another nut, drops another egg, and so continues till she has exhausted her entire stock. The young nut, not essentially injured, continues to grow. The egg is soon hatched; the young larve, or maggot, finds its food already ripened and in waiting for it; and, about the time of its full growth, falls, with the mature nut, to the ground, and at length creeps out by gnawing a circular hole in its side. It then burrows under the surface of the ground, where it continues dormant for eight months; at the termination of which time it casts its skin, commences a chrysalis of the general shape and appearance of the beetle kind, and, in the beginning of August, throws off the chrysalid investment, creeps to the surface of the ground, finds itself accommodated with wings, becomes an inhabitant of the air, and instantly pursues the very same train of actions to provide for a new progeny which had been pursued by the parent-insect of the year before.

The instances are innumerable: let us, however, confine ourselves to one more. The sphex, or ichneumon wasp, in its perfect state, feeds on the nectary of flowers; but, as soon as she is fitted to deposit her eggs, she becomes actuated by an appetite of another kind. She first bores a small cylindrical hole in a sandy soil, into which, by accurately turning round and adjusting herself like the nut-weevil, she also drops an egg. She next seeks out a small green caterpillar inhabiting the leaves of the cabbage-plant, which she punctures with her sting, yet so slightly and delicately as not to kill it; she then rolls it up into a circle, and places it in the sandy nest, immediately over the egg. She continues the same labour till she has counted twelve, and deposited twelve caterpillars, one over another; and thus repeats the process of ovation and supply, till she has exhausted herself of her entire stock of eggs. She immediately closes the hole, and dies; entrusting her eggs to the fostering heat of the sun. The egg in each separate cell or aperture is soon hatched, and the young larve finds, in the superincumbent green caterpillar, its food duly prepared and in waiting for it, and incapable, from its enfeebled state, of resisting its attack; though preserved from putrefaction by the little life that yet remains to it. It feeds progressively on the twelve caterpillars, and, by the time it has exhausted them, becomes fitted for, and is converted into, a chrysalis. In the proper season it awakes from its dormancy, works its way to the surface of the earth, throws off its chrysalid clothing, employs its fresh wings with dexterity, rises into the new element of the atmosphere, feeds on the honey of plants instead of on maggots; and at length pursues the very same train of actions to provide itself with a progeny, which was pursued by the parent-insect that gave it a being.

To what train of thought or feeling,—to what description of habit, imitation, or experience, can we possibly ascribe such wonderful and uniform series of actions in beings whose generation is equally estranged to that which precedes and that which follows them? that only live after the death of their parent, and die before the birth of their progeny? In all such cases it is clear that there is a principle implanted in the living form, equally distinct from all mechanical, chemical, and inductive or reasoning powers, which, through the nice labyrinth of its conduct, directs the agent by an unerring impulse; or, in other words, impels it by a prescribed and unerring law, to accomplish a definite end by a definite mean.

Instinct, then, may exist separately from both perception and sensation. I will add to this imperfect sketch a few examples, in which it will be obvious to every one that it may exist also in conjunction with each of these principles.

And first as to its union with sensation. Wherever a nervous system is to be traced, which alone is the source of sensation, we have abundant proofs of such an alliance. We meet with it, without having any adequate language by which to describe it, in the pleasurable glow and elasticity of health,—in the satisfaction of a cheerful meal,—in the painful sense of fatigue,—and in the refreshment of sound and natural sleep, by which this painful sense is overcome; all which some of the German physiologists have faintly attempted to express by the terms *selbstgefühl*, SELF-FEELING, and *gemeingefühl*, GENERAL FEELING.* And we meet with it still more strongly marked, and in diversities which no terms have been strong enough to delineate in any language, in all those natural emotions of love of life and dread of death, the economy of pairing and attachment to offspring, which, in consequence of such alliance, have obtained, as already noticed, the popular name of instinctive sensations. Let us select a few examples.

* HUBNER, *Comment. de Cœnesthesi, &c.* 1794.

We are told by Galen,* that, on opening a goat big with young, he found one of the young ones alive, which he hastily snatched up, and took into a room where there were various vessels severally filled for the purpose with wine, oil, honey, milk, grains, and fruits. The little kid first rose upon its feet, and walked; then shook itself, and scratched its side with one of its hoofs; it next smelt, in succession, at all the dishes before it, and at last fixt upon and licked up the milk. In this case, the sense of smell went directly in aid of the instinctive search after food, and determined the particular kind. So that the instinct and the sensation co-operated.

Thus rabbits, when left to the operation of pure instinct, dig holes in the ground for warmth and protection; but, after being for some time in a domestic state, and finding that they can obtain a more comfortable asylum by other means and with less labour, they seldom pursue, even when they have an opportunity, the instinctive process, but burrow in the straw, or whatever other material is provided for them. In this case, the sense of superior comfort combines itself, as in the preceding case, with the operative instinct, and the same end is pursued, though by a change of means.

So, again, the young of all animals, in whatever way they take their food, are at first stimulated by instinct alone. The lamb sucks, the chicken pecks, and the nestling of the sparrow gapes. In like manner, the mother at first secretes or selects it from an instinctive excitement alone. The udder of the dam swells and becomes painful; the crop of the pigeon does the same; and there are some birds, whose common food is grain, that during this season devour for their young spiders and other insects, which nothing could induce them to touch at any other time. What follows from this simple exercise of instinctive power, is well worthy of notice. This sweet intercourse of natural action soon lays a foundation for a something that, by degrees, shows itself to be superior to simple instinct, though it has often, but erroneously, been so denominated. The young of two different mothers, if interchanged as soon as they are born or hatched, are as satisfied with the foster, or supposititious, as with the natural parent: and the mothers, unless made suspicious of the deception, are as satisfied with their foster or supposititious young. But let the same interchange be attempted a month or two afterwards, and in no case will it succeed. Short as has been the intervening period, it has sufficed to produce a birth of feeling, as well as a growth of form; the rising sense has united itself with the already mature instinct; and the natural nurse, and the natural nurseling, will pine equally, if

* *De Locis,* lib. vi. cap. 6.

separated from each other. The mere instinct of this early age belongs to such early age alone, and to the purpose of such early age alone: and when it has answered that purpose, it ceases, and we meet with no more trace of it; but the feeling which follows so close upon it, and to which it may in some sort be said to have given birth, is of a higher order, and continues for a much longer period of time; and for a period of time, indeed, directly proportioned to its intensity,—or, in other words, to the ascending rank of sentient or percipient life in which it makes its appearance.

Hence, in the two lowest classes of animals, we meet with nothing of the sort whatever; the young of insects and worms having a foreign food provided for them, without the immediate intervention of the mother: and hence too, in quadrupeds and birds, the feeling progressively dies away, as the young become independent: while, in man, we behold the principle of intelligence in its most lovely and interesting character,—a moral and internal feeling,—a sense of gratitude and veneration on the one side, of keen complacency and delight on the other, and of active affection on both,—catching hold of the two preceding principles, and producing a strong cord of interunion, that can never be broken but with the cords of the heart itself. Something of the kind is occasionally, indeed, to be met with in quadrupeds, as in the seal and the lumantin (tricheus *manatus)*, which pass through life in families of single male and single female, never deserting or deserted by their young, till the latter, having reached the term of maturity, separate to found families of their own.

In these cases, we see examples of all the three principles of instinct, sensation, and intelligence, in a state of union; and we occasionally meet with still more extraordinary examples of the same fact. One of the most extraordinary, perhaps, is that related by Mr. Gilbert White, in his very interesting "History of Selborne," of the gratitude and affection of a young hare towards a cat, by which it had been suckled and brought up; the leveret following the cat about the garden, playing with her like a kitten, and bounding towards her when purring or uttering any other call of tenderness.

We see also something of the same kind of internal feeling, and often exalted to a still higher pitch, in the gratitude and affection of the fond and faithful dog for a kind and indulgent master; occasionally, indeed, rising superior to, and strikingly triumphing over, the strongest instinctive feelings of the animal frame,—over thirst, hunger, and the love of life itself; and inciting him to perish voluntarily by the side of his master, and share his grave, rather than abandon his corse, when, in the course of a solitary journey, he has suddenly fallen a victim to accident or violence. Of this the late Bishop of Landaff, in his very interesting Life, has noticed a very singular example; who tells us that, two months after the disappearance of a man that had perished on the top of Helvellyn, his body was found, and his faithful dog was still sitting by it: and to this he adds an anecdote told him by the Duke of Northumberland, concerning a young antelope that had died by a fall, whose mother immediately quitted the pasture in which she was feeding, sat piteously by the side of the body, which she refused to quit, and died herself of grief and hunger.

And not unfrequently we meet with instances of the union of intelligence alone with instinct alone, no moral or internal feeling being necessary. The rook usually and instinctively builds his nest in the tallest branches of the tallest trees; but in Welbourn church-yard, as we learn by a letter to Dr. Darwin from a relation,* a rookery was not long since formed on the outside of the church-spire, and the tops of its loftiest windows. There had formerly been a row or grove of trees in the neighbourhood; but they had been cut down, and their aerial tenants, being thus dispossest of their proper mansion, had betaken themselves to the church-spire and windows, as the most appropriate building for their purpose; and had thus manifestly evinced an alliance of instinct with intelligence,—with contrivance suited to the occasion.

So the jack-daws of Selburn, according to Mr. White, not finding a sufficiency of towers and steeples, and lofty houses, on which they had usually hung their nests in this pleasant village, accommodated themselves to the occasion, and built them in forsaken rabbit-burrows.

The ostrich is accused of a total want of natural feeling, because she abandons her eggs to be hatched by the heat of the sun. Where incubation is necessary, however, the ostrich instinctively employs it; and that, too, in conjunction with an intelligence which is rarely evinced by other birds. Thus, in Senegal, where the heat is still great, she relinquishes her eggs during the day, but sits upon them through the chill of the night; and at the Cape of Good Hope, where the heat is less considerable, she sits upon them, like other birds, both day and night. The cuckow, in like manner, is well known to be too indolent to build where she can obtain a nest ready-made, and usually deposits her eggs in that of the hedge-sparrow; but, where no proper nest can be procured already formed for her use, she constructs for herself, and incubates her young. In like manner, ducks and geese, though not renowned for sagacity, cover up their eggs when they quit them, till their return to the

* *Zoonom.* i. p. 241, 8vo. edit.

nest; and there are few birds that do not turn and shift their eggs at different periods of the tedious process of incubation, so as to give an equal degree of warmth to every part.

There are various instincts, mostly connected with a peculiar configuration, which to us are altogether anomalous, though highly worthy of notice. But they at least show that the great Author of Nature is the lord, and not the slave of his own laws, and is at all times capable of producing definite effects by a diversity of means. The didus *solitarius*, or solitary dodo, in general esteemed almost as stupid a bird as the ostrich, divides the labour of incubation with his female, and sits upon the eggs during her absence. The hen of this tribe has a protuberance on each side of the breast, like the teat of quadrupeds. When the young of the turtle-dove are hatched, and capable of receiving nutriment from the crop of the mother, the male parent experiences an equal change and enlargement in this organ, secretes the same nutritive material, and equally contributes to the support of its nestlings.

I have already observed, that insects in general deposit their eggs in places admirably suited to the future wants of the nascent larves, and then for ever take leave of their embryon-progeny; but the forficula *auricularia*, or common ear-wig, broods over her young like a hen, and only quits them at night, the usual period in which this genus flies in pursuit of food or recreation.

Among migratory birds, it is not very uncommon for the males alone to dare the dangers of a distant voyage, and to leave the females behind them; but, in the fringilla *cœlebs*, or chaffinch, we occasionally find this rule inverted: for the female chaffinches of Sweden quit their males, and migrate to Holland towards the winter, and duly return to them in the spring; while many of the males indulge a profound sleep during the greater period of their absence.— husbands comfortable.

Most vegetables indulge in a winter-sleep of the same kind, but there are some that sleep still longer. Thus the tuberose root of the ferraria, an ornamental herbaceous plant of the Cape of Good Hope, remains torpid every alternate year, and sometimes continues in this state for two years together, without putting forth either leaf or fibre.

The general corollary, then, resulting from these observations, is as follows: that INSTINCT, as already defined, is the operation of the principle of organised life, by the exercise of certain natural powers directed to the present or the future good of the individual; while reason is the operation of the principle of intellectual life, by the exercise of certain acquired powers directed to the same object. That it appertains to the whole organised mass, as gravitation does to the whole unorganised; equally actuating the smallest and the largest portions, the minutest particles and the bulkiest systems, every organ, and every part of every organ, whether solid or fluid, so long as it continues alive. That, like gravitation, it exhibits, under particular circumstances, different modifications, different powers, and different effects; but that, like gravitation too, it is subject to its own peculiar division of laws, to which, under definite circumstances, it adheres without the smallest deviation; and that its sole and uniform aim, whether acting generally or locally, is that of perfection, preservation, or reproduction.

Of its mode of existence, we know nothing; but as little do we know of the principle of gravitation or of mind. We can only assure ourselves that they are distinct powers,—perhaps distinct essences; and we see them acting, as well separately as conjointly, for the general good. Under their accordant influence, we behold the plastic and mysterious substance of matter, which we must be especially careful not to confound with themselves, rising from "airy nothing" into entity,—ascending from invisible elements into worlds, and systems of worlds,—from shapeless chaos and confusion into form, and order and harmony,—from brute and lifeless immobility into energy and activity, into a display of instinct, feeling, perception,—of being, and beauty, and happiness. One common design, one uniform code of laws, in different but consenting divisions, equally simple and majestic, equally local and comprehensive, pervades, informs, unites, and consummates the whole.

The effect, then, being one, the MIGHTY CAUSE that produced it must be one also: an eternal and infinite unity,—the radiating fountain of all possible perfections,—ever active, but ever at rest,—ever present, though never seen,—immaterial, incorporeal, ineffable; but the source of all matter, of all mind, of all existence, and all modes of existences. Whatever we behold is GOD: all nature is his awful temple,—all sciences the porticoes that lead to it, and the chief duty of philosophy is to conduct us to his altar; to render all our attainments, which are but the bounteous afflations of his spirit, subservient to his glory; and to engrave on the tablet of our hearts this great accordant motto of all natural and of all revealed religion, of Athens and of Antioch, of Aratus and of St. Paul,—"IN HIM WE LIVE, AND MOVE, AND HAVE OUR BEING."

